# Impact of an Extraglottic Device on Pediatric Airway Management in an Urban Prehospital System

**DOI:** 10.5811/westjem.2019.8.44464

**Published:** 2019-10-21

**Authors:** Daniel G. Ostermayer, Elizabeth A. Camp, James R. Langabeer, Charles A. Brown, Juan Mondragon, David E. Persse, Manish I. Shah

**Affiliations:** * McGovern Medical School, University of Texas Health Sciences Center, Department of Emergency Medicine, Houston, Texas; † Baylor College of Medicine, Texas Children’s Hospital, Department of Pediatrics, Section of Emergency Medicine, Houston, Texas; ‡ McGovern Medical School, University of Texas Health Sciences Center, Department of Emergency Medicine, Houston, Texas; § University of Michigan Medical School, Department of Emergency Medicine, Ann Arbor, Michigan; ¶ Houston Fire Department, Houston, Texas

## Abstract

**Introduction:**

Prehospital pediatric endotracheal intubation has lower first-pass success rates compared to adult intubations and in general may not offer a survival benefit. Increasingly, emergency medical services (EMS) systems are deploying prehospital extraglottic airways (EGA) for primary pediatric airway management, yet little is known about their efficacy. We evaluated the impact of a pediatric prehospital airway management protocol change, inclusive of EGAs, on airway management and patient outcomes in children in cardiac arrest or respiratory failure.

**Methods:**

Using data from a large, metropolitan, fire-based EMS service, we performed an observational study of pediatric patients with respiratory failure or cardiac arrest who were transported by EMS before and after implementation of an evidence-based airway management protocol inclusive of the addition of the EGA. The primary outcome was change in frequency of intubation attempts when paired with an initial EGA. Secondary outcomes included EGA and intubation success rates and patient survival to hospitalization and discharge.

**Results:**

We included 265 patients age <16 years old, with 142 pre- and 123 post-protocol change. Patient demographics and event characteristics were similar between groups. Intubation attempts declined from 79.6% pre- to 44.7% (p<0.01) post-protocol change. In patients with an intubation attempt, overall intubation success declined from 81.4% to 63.6% (p<0.01). Post-protocol change, an EGA was attempted in 52.8% of patients with 95.4% success.

**Conclusion:**

Implementation of an evidenced-based airway management algorithm for pediatric patients, inclusive of an EGA device for all age groups, was associated with fewer prehospital intubations. Intubation success may be negatively impacted due to decreases in procedural frequency.

## INTRODUCTION

### Background

Prior research suggests the addition of paramedic endotracheal intubation (ETI) in pediatric patients does not improve survival or neurologic outcomes in children.^1^ Median success rates for prehospital ETI in the United States are lower than those for extraglottic airway (EGA) placement.^2^ Currently, the national emergency medical services (EMS) educational standards for paramedics do not define intubation training requirements for paramedics.^3^ Also, paramedics have few requirements during training to adequately practice the skill of intubation,^4^ and few ongoing opportunities to maintain proficiency.^4^–^5^,^6^ Neonatal resuscitations that use EGAs have demonstrated safety, high placement success, and improved resuscitation rates when compared to bag-valve mask ventilation (BVM).^7^ Limited data exists across the entire pediatric age spectrum on the use of EGAs, especially in EMS.

**Figure 1:**
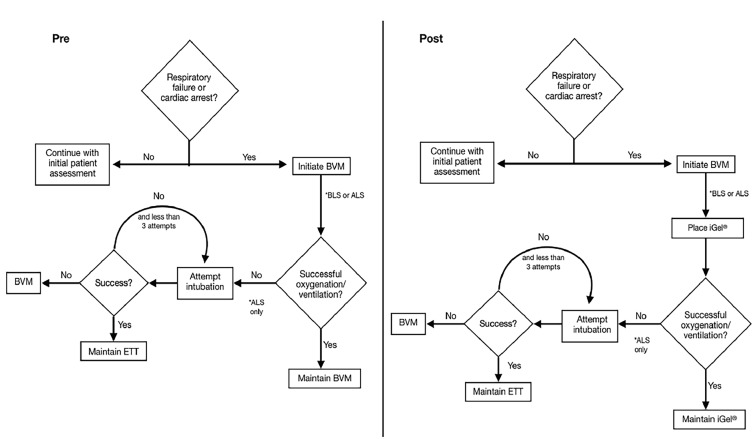
Post-intervention airway management algorithm. *ETT*, endotracheal tube; *BLS*, basic life support; *ALS*, advanced life support; *BVM*, bag valve mask; *iGel*, supraglottic airway device from Intersurgical.

A National Association of EMS Physicians position statement recommends that EMS have at least one blindly inserted nonsurgical airway available.^8^ Likewise, the American Academy of Pediatrics Committee on Pediatric Emergency Medicine and the American College of Emergency Physicians Pediatrics Committee have recommended the inclusion of EGAs with supplies for difficult airway conditions in the emergency department.^9^ In 2014 the National Association of State EMS Officials (NASEMSO) published its Model Clinical EMS Guidelines, which included recommendations from an evidence-based guideline for pediatric airway management that was implemented as part of a separate project in several New England states and the City of Houston Fire Department (HFD). The guideline emphasized step-wise escalation in airway management from BVM to EGA to ETI, only if the less-invasive method was not effective (Figure 1).^10^

**Population Health Research Capsule**

What do we already know about this issue?

*Extraglottic airways have high procedural success and increasing deployment in EMS systems for pediatric airway management*.

What was the research question?

Does widespread deployment of an extraglottic airway affect frequency of intubation in pediatric patients with respiratory failure or cardiac arrest?

What was the major finding of the study?

*Increased use of an extraglottic airway by EMS for pediatric airway management resulted in fewer intubations, potentially affecting procedural success with intubation*.

How does this improve population health?

*EMS systems using extraglottic airways and intubation should continue intensive airway management education with all available devices to maintain procedural competency*.

Our study evaluated the impact of a pediatric prehospital airway management-protocol change consistent with the NASEMSO guidelines and inclusive of a pediatric EGA, on airway management and patient outcomes in children with prehospital respiratory failure.

## METHODS

We performed a retrospective cohort study of pediatric patients <16 years old cared for by the HFD EMS from January 1, 2013 – March 31, 2017. We compared the intubation rates, operational metrics, and clinical outcomes of pediatric patients with respiratory failure (respiratory rate < 5 breaths per minute or oxygen saturation <85%) or in cardiac arrest two years before and after an airway management algorithm (Table 1) change that included addition and prioritization of the EGA device, i-gel, (Intersurgical Ltd., Berkshire, UK). We used recorded end-tidal waveform capnography as a marker of both EGA and endotracheal tube success, or paramedic-reported passage through the vocal cords for ETI success. Prehospital return of spontaneous circulation (ROSC), as recorded from the patient care and records, was defined as presence of a pulse with cessation of cardiopulmonary resuscitation (CPR) prior to hospital arrival. We recorded survival outcomes from both hospital records and the EMS agency cardiac arrest database.

### Study Setting

HFD is a two-tiered 9-1-1 EMS system with Basic Life Support (BLS) and Advanced Life Support (ALS) units. HFD serves a geographic area totaling 2.3 million persons and 667 square miles in the greater Houston region. The agency receives 300,000 EMS calls annually. No other EMS agencies provide emergency 9-1-1 response within Houston city limits. HFD has 3500 prehospital providers, all of whom are trained as firefighters and have at least BLS emergency medical technician (EMT) training. HFD also has 700 paramedics providing ALS care. Dispatch of the initial unit is determined based on the 9-1-1 call type and severity.^11^ The local EMS protocol for management of respiratory failure in pediatric patients changed to include the use of an EGA for pediatric patients – the i-gel – in addition to algorithmic progression from one device to a more advanced device. Prior to the protocol change no EGA device was available for pediatric airway management due to the size restrictions of the then-used King LT-D airway (Ambu, Copenhagen, Denmark).

Prior to the protocol change pediatric patients with respiratory failure or cardiac arrest were managed first with BVM followed by intubation. Both ALS and BLS providers were equipped with the i-gel EGA post-protocol change for both adults and pediatric patients. The King LT-D was not available post-protocol change. The airway management protocol directed members to use BVM first and then advance to an EGA for all patients requiring transport and continued assisted ventilation. If the EGA provided inadequate oxygenation or ventilation it could be removed, with intubation attempted by a paramedic. The new protocol inclusive of EGAs was implemented in conjunction with an in-person lecture and skills training described in a prior publication.^12^ No other aspects of pediatric cardiac arrest management changed during the study period. All study patients received ALS care.

### Data Collection

**Figure 2:**
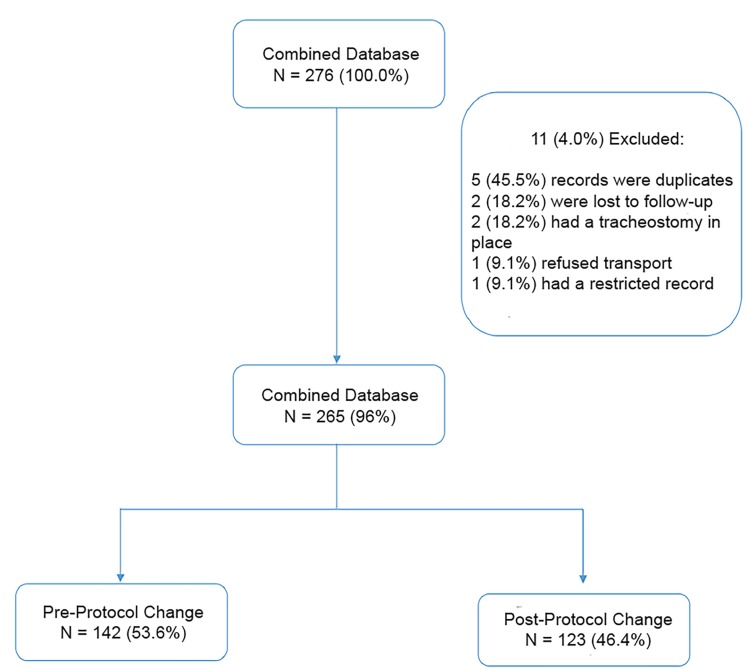
Patient flow diagram for before and after analysis of implementation of new prehospital pediatric airway management process incorporating supraglottic ariway.

We retrospectively reviewed electronic patient data to establish the baseline characteristics, incidence of airway procedures, and outcomes for patients meeting this study’s inclusion criteria (Figure 2). Prospective patients were electronically identified on a weekly basis via the patient care record (Imagetrend, Lakeville, MN) and cardiac arrest quality-assurance databases. Records were reviewed by trained abstractors (CB, JM) who were aware of the study design and outcomes in question. Hospital and outcome data were abstracted from the EMS agency’s cardiac arrest database and hospital inpatient medical records.

### Statistical Analysis

Our primary outcome was a difference in the frequency of prehospital attempted intubations between the pre- and post-intervention groups. We estimated a 20% reduction in intubation rate from implementation of the new protocol with a sample size of 266 (alpha 0.05, power 0.8). For skewed continuous data (Shapiro-Wilks<0.001) we used non-parametric testing (Mann-Whitney test). Incomplete data or negative timed operational metrics (ie, time on scene) were coded as missing. We analyzed categorical variables using the Pearson chi-square test or Fisher’s exact test. A p-value less than 0.05 was considered statistically significant. Categorical variables were reported using frequencies and percentages, continuous variables were reported using median and interquartile ranges. We conducted all analyses using the Statistical Package for the Social Sciences (SPSS), version 24 (IBM Corp., Armonk, NY).

### Institutional Review Board Approvals

The University of Texas Health Science Center at Houston and Baylor College of Medicine institutional review boards approved this study. The study was approved with waiver of consent for patients observed and data accessed.

## RESULTS

Demographically and clinically, there were no significant differences between patients during the pre- and post-protocol change timeframes (Table 1). We found a significant difference in the frequency of intubation and the success of intubation in the two groups. Specifically, the number of children with an ETI attempted decreased from 79.6% pre to 44.7% post (p<.001). In those that had ETI attempted, the overall success rate was 81.4 % pre and 63.6% post (p<.001). Post protocol, 52.8% had an attempted EGA airway with a success rate of 95.4%. Table 2 summarizes the number of advanced airway attempts. The majority of patients pre and post, 74.6% and 66.7% respectively, were in cardiac arrest (Table 3).

**Figure 3:**
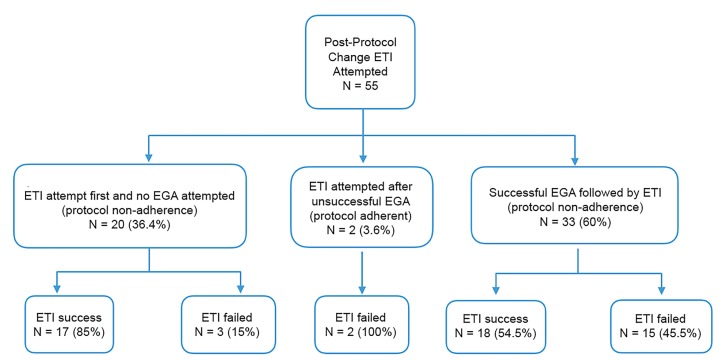
Endotracheal intubations post-protocol change.

Of the intubations attempted after the protocol change, 96.4% were not performed in adherence to the protocol change since 36.4% had no EGA attempted and 60% had intubation performed after successful EGA placement (Figure 3). The vast majority of patients during this period were in cardiac arrest (Table 3) with no difference between pre and post with regard to initial arrest rhythm. Our study was not powered to detect a prehospital ROSC or survival benefit in cardiac arrest patients,^13^ and we did not find a significant change in prehospital ROSC (Table 3) or survival to hospital admission or discharge (Tables 2 and 3).

## DISCUSSION

In this observational study, we found that the establishment of an airway management algorithm paired with an EGA suitable for all ages of pediatric patients decreased the rate of ETI in an urban EMS system. No differences in survival to hospital admission or discharge were observed in all patients with cardiac arrest or respiratory failure. For cardiac arrest patients specifically, we observed no difference in rates of ROSC. These observations suggest that deployment of a pediatric EGA can successfully decrease the need for prehospital intubation.

Although prior research suggests no improvement in neurologic outcome with ETI,^1^ the skill is taught as part of the EMT-Paramedic National Standard Curriculum and still widely practiced in EMS agencies across the U.S.^14^ As many EMS agencies progress toward widespread EGA deployment given evidence against significant benefits from intubation during initial cardiac arrest care, intubation skill retention remains largely unknown.^15^,^16^ For pediatric patients especially, the effects of implementing an EGA-first strategy decreases a paramedic’s exposure to the already rare intubation. Prior research has demonstrated a low number of clinical opportunities for paramedics to maintain procedural competency with intubation,^5^ let alone the exceedingly rare pediatric intubation.

In our cohort, we observed a decline in the success rate for pediatric intubations when attempted (81.4% vs 63.6%) after introducing an EGA. The effects of implementing the EGA in this system, while continuing to allow ETI, resulted in a further dilution of procedural experience. The potential difficulty with maintaining paramedic intubation skills for pediatric and adult patients, is well documented by prior studies,^5^,^17^–^20^ and may be augmented in systems such as this where ETI exists concurrently with EGA prioritization. The potential training solutions and their effectiveness have not been described. High-performance EMS agencies with intensive training, continuing education, and quality assurance report intubation success rates as great as 97% but with low first-pass success.^17^ Systems with infrequent airway management training and skill maintenance when coupled with the addition and widespread use of EGAs may experience declines in success, as those observed in our system.

However, in the intubations that occurred post-protocol change, 96.4% occurred due to protocol non-adherence. Despite our reported 95% success rate with EGA placement, which is consistent with previous publications,^21^,^22^ many patients during the study period still underwent ETI attempts. Of the 36.4% with ETI attempted prior to an EGA attempt only, 85% experienced a success. Similarly, only 54.4% were successful when attempted after an already successful EGA. Although prior commentary has suggested that EGAs, specifically the i-gel, perform well in the prehospital environment, success rates may be lower than previously demonstrated in hospital-based studies.^21^,^23^

In non-paralyzed adults, for example, ventilation with the adult size 4 i-gel may exceed the 24 millimeters of mercury laryngeal seal, causing significant air leak.^24^ For children, the degree of leak if the device is sized incorrectly is unknown. For our cohort, the rationales behind the protocol deviations (Figure 3) were not consistently documented. It is possible that many of the ETIs after EGA placement were in fact warranted but appeared as protocol violation due to inadequate documentation of EGA failure. Providers’ perception of inadequate ventilation or incorrect device sizing may have contributed to the intubation attempts occurring after initial EGA placement.

Our study was not powered to detect a prehospital ROSC or survival benefit in cardiac arrest patients.^13^ In this small cohort we did not observe any measurable effects on cardiac arrest care, although metrics such as compression fraction, CPR rate, and exact timing of EGA or ETI were not available. Also, given our small sample size and low frequency of shockable rhythms in the pediatric population,^25^ further research is required to address the initial airway management device by rhythm and likelihood of a primary respiratory arrest.^17^,^26^

## LIMITATIONS

This study is not without limitations. First, it was partially limited by the nature of the retrospective review that determined the EMS system’s baseline in addition to the inability to associate clinical outcomes with the applied airway device. The small cardiac-arrest subset also limits generalizability to pediatric cardiac care. There was also unclear documentation with regard to paramedic reasoning to proceed through the airway algorithm to a more advanced device. Further, some patients’ airways were managed with multiple devices or the same device multiple times in different sizes. We could not analyze this dataset for correlation with weight and device sizing. In addition, success was based on provider documentation rather than direct review of capnography waveforms. Due to limitations with record review, isolating the effect of a singular airway management device or timing of placement was not possible.

## CONCLUSION

Implementation of an evidenced-based airway management algorithm for pediatric patients paired with EGA devices for all ages was associated with decreased frequency of prehospital pediatric intubation. Intubation success when attempted may be negatively impacted by the decrease in skill frequency.

## Figures and Tables

**Table 1:** Baseline characteristics of patients before and after a change in the airway management protocol.

	Pre-protocol change N = 142 (53.6%) N (%) or Median (IQR)	Post-protocol change N = 123 (46.4%) N (%) or Median (IQR)	P-value
Age (years)	1.0 (0,6)	1.2 (0,6)	0.79
Sex			0.76
Female	58 (40.8)	48 (39.0)	
Male	84 (59.2)	75 (61.0)	
Race			0.41
Hispanic	61 (43.0)	47 (38.2)	
Caucasian	15 (10.6)	9 (7.3)	
African American	59 (41.5)	63 (51.2)	
Other	7 (4.9)	4 (3.3)	
Top paramedic working assessments			0.05
Cardiac	114 (80.3)	87 (70.7)	
Respiratory	10 (7.0)	11 (8.9)	
Seizure	5 (3.5)	10 (8.1)	
Trauma	3 (2.1)	10 (8.1)	
Other	10 (7.0)	5 (4.1)	
Traumatic arrest	16 (11.3)	14 (11.4)	0.74
ALS on scene time (minutes)*	27.0 (18, 36)	24.0 (18, 34)	0.17

* N=10 missing scene time pre and 13 post.

*IQR*, interquartile range; *ALS*, Advanced Life Support.

**Table 2:** Airway interventions and outcomes for all patients pre- and post-airway management change.

	Pre-protocol change N = 142 (53.6%) N (%) or Median (IQR)	Post-protocol change N = 123 (46.4%) N (%) or Median (IQR)	P-value
ETI attempted	113 (79.6)	55 (44.7)	<0.001
Intubation success	92 (81.4)	35 (63.6)	<0.001
ETI attempts if successful	1.0 (1.0, 2.0)	1.0 (1.0, 2.0)	0.36
ETI attempts if intubation unsuccessful	2.0 (1.0, 3.0)	1.50 (1.0, 2.0)	0.22
EGA attempted	N/A*	65 (52.8)	N/A
EGA success	N/A*	62 (95.4)	N/A
Survival to hospital admission	50 (35.2)	49 (39.8)	0.44
Survival to hospital discharge	30 (21.1)	31 (25.2)	0.38

* Extraglottic airways were not part of the pediatric protocol during the pre-protocol change period.

*IQR*, interquartile range; *ETI*, endotracheal intubation; *EGA*, extraglottic airway.

**Table 3:** Cardiac arrest subgroup.

	Pre-protocol change* N = 106 (56.3%) N (%) or Median (IQR)	Post-protocol change* N = 82 (43.7%) N (%) or Median (IQR)	P-value
Bystander CPR	39 (36.8)	42 (51.2)	0.048
Witnessed arrest	31 (29.2)	22 (26.8)	0.72
VF/VT	4 (3.8)	2 (2.4)	0.70
PEA	19 (17.9)	15 (18.3)	0.95
Asystole	78 (73.6)	63 (76.8)	0.61
Undocumented rhythm	5 (4.7)	2 (2.4)	0.47
ROSC	25 (23.6)	17 (20.7)	0.64
Survival to hospital admission	28 (26.4)	17 (20.7)	0.37
Survival to hospital discharge	11 (10.4)	7 (8.5)	0.67

* P-value was calculated using Fisher’s exact test when any cell value was less than five.

*IQR*, interquartile range; *CPR*, cardiopulmonary resuscitation; *VF*, ventricular fibrillation; *VT*, ventricular tachycardia; *PEA*, pulseless electrical activity; *ROSC*, return of spontaneous circulation.

## References

[1] Gausche M, Lewis RJ, Stratton SJ (2000). Effect of out-of-hospital pediatric endotracheal intubation on survival and neurological outcome: a controlled clinical trial. JAMA.

[2] Wang HE, Mann NC, Mears G (2011). Out-of-hospital airway management in the United States. Resuscitation.

[3] US Department of Transportation NHTSA (2009). National Emergency Medical Services Education Standards: Paramedic Instructional Guidelines. EmsGov.

[4] National Highway Traffic Safety Administration Emergency Medical Technician Paramedic: National Standard Curriculum (EMT-P).

[5] Wang HE, Kupas DF, Hostler D (2005). Procedural experience with out-of-hospital endotracheal intubation. Crit Care Med.

[6] Burton JH, Baumann MR, Maoz T (2003). Endotracheal intubation in a rural EMS state: procedure utilization and impact of skills maintenance guidelines. Prehospital Emerg Care.

[7] Zhu XY, Lin BC, Zhang QS (2011). A prospective evaluation of the efficacy of the laryngeal mask airway during neonatal resuscitation. Resuscitation.

[8] O’Connor RE (2007). Alternate airways in the out-of-hospital setting position statement of the National Association of EMS Physicians. Prehospital Emerg Care.

[9] (2013). Joint policy statement: Guidelines for care of children in the emergency department. J Emerg Nurs.

[10] National Association of State EMS Officials National Model EMS Guidelines version 2.2.

[11] Persse DE, Key CB, Bradley RN (2003). Cardiac arrest survival as a function of ambulance deployment strategy in a large urban emergency medical services system. Resuscitation.

[12] Marino MC, Ostermayer DG, Mondragon JA (2018). Improving prehospital protocol adherence using bundled educational interventions. Prehospital Emerg Care.

[13] Hasegawa K, Hiraide A, Chang Y (2013). Association of prehospital advanced airway management with neurologic outcome and survival in patients with out-of-hospital cardiac arrest. JAMA.

[14] Hansen M, Loker W, Warden C (2016). Geospatial analysis of pediatric EMS run density and endotracheal intubation. West J Emerg Med.

[15] Benger JR, Kirby K, Black S (2018). Effect of a strategy of a supraglottic airway device vs tracheal intubation during out-of-hospital cardiac arrest on functional outcome: the AIRWAYS-2 randomized clinical trial. JAMA.

[16] Wang HE, Schmicker RH, Daya MR (2018). Effect of a strategy of initial laryngeal tube insertion vs endotracheal intubation on 72-hour survival in adults with out-of-hospital cardiac arrest: a randomized clinical trial. JAMA.

[17] Prekker ME, Delgado F, Shin J (2016). Pediatric intubation by paramedics in a large emergency medical services system: process, challenges, and outcomes. Ann Emerg Med.

[18] Carlson JN, Gannon E, Clay Mann N (2015). Pediatric out-of-hospital critical procedures in the United States. Pediatr Crit Care Med.

[19] Hansen M, Lambert W, Guise JM (2015). Out-of-hospital pediatric airway management in the United States. Resuscitation.

[20] Dyson K, Bray JE, Smith K (2017). Paramedic Intubation experience is associated with successful tube placement but not cardiac arrest survival. Ann Emerg Med.

[21] Thomas M, Benger J (2009). Pre-hospital resuscitation using the iGEL. Resuscitation.

[22] Gabbott DA, Beringer R (2007). The iGEL supraglottic airway: a potential role for resuscitation?. Resuscitation.

[23] Duckett J, Fell P, Han K (2014). Introduction of the i-gel supraglottic airway device for prehospital airway management in a UK ambulance service. Emerg Med J.

[24] Gatward JJ, Cook TM, Seller C (2008). Evaluation of the size 4 i-gel^TM^ airway in one hundred non-paralysed patients. Anaesthesia.

[25] Schindler MB, Bohn D, Cox PN (1996). Outcome of out-of-hospital cardiac or respiratory arrest in children. N Engl J Med.

[26] Young KD, Seidel JS (1999). Pediatric cardiopulmonary resuscitation: a collective review. Ann Emerg Med.

